# Effective robotic arm-assisted traction for endoscopic submucosal dissection treating ulcerative colitis-associated colonic neoplasia

**DOI:** 10.1055/a-2851-8432

**Published:** 2026-05-13

**Authors:** Norio Fukami, Daisuke Yamaguchi, Ibrahim H. Hassan, Hyun Jae Kim, Mitsuru Esaki

**Affiliations:** 1 Department of Gastroenterology and Hepatology 23387 Mayo Clinic ArizonaScottsdale, ArizonaUnited States; 2 Division of Gastroenterology, Department of Internal Medicine, Faculty of MedicineSaga UniversitySagaJapan

Ulcerative colitis (UC) is associated with an increased risk of colorectal neoplasia, and endoscopic submucosal dissection (ESD) can be challenging in UC because chronic inflammation and submucosal fibrosis often result in poor lifting and an unstable dissection plane. ROBOPERA (EndoRobotics, Seoul, Korea) is a robotic arm-assisted ESD platform that enables multidirectional traction against gravity 1 . Robotic arm-assisted ESD has been reported to improve submucosal exposure and procedural efficiency 2 3 4 .

**Fig. 1:**
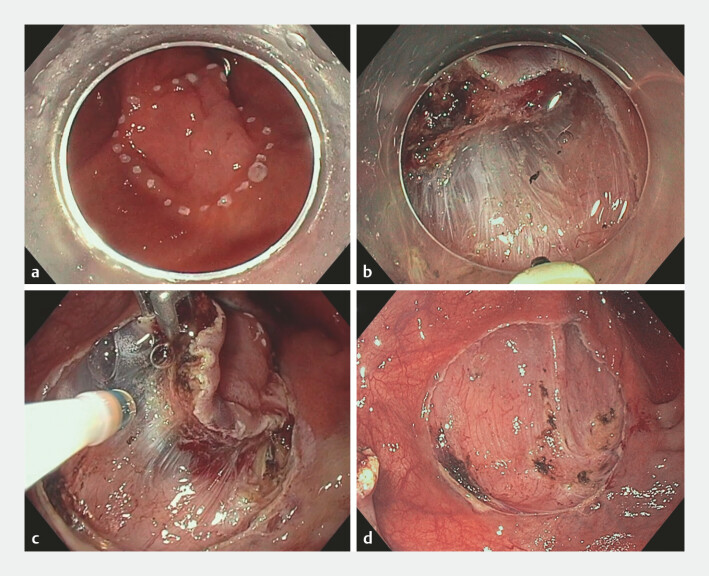
**a** A 20-mm flat lesion in the proximal descending colon. **b** Submucosal fibrosis during ESD. **c** Lesion traction using robotic arm assistance. **d** En bloc resection achieved by ESD. ESD, endoscopic submucosal dissection.

A 60-year-old man with long-standing UC in clinical remission (Mayo endoscopic subscore 0) was found to have a 20-mm flat lesion in the proximal descending colon ( Fig. 1 **a** ). Biopsy and distal tattooing were performed, and pathology suggested a sessile serrated lesion with dysplasia. The patient was referred for endoscopic resection and colonoscopy with ESD was planned.

A 20-mm lesion (Paris classification IIa) was identified proximal to the tattoo. Narrow-band imaging suggested a serrated change with focal area of dysplasia ( Video 1 ). After circumferential marking and submucosal injection (saline, methylcellulose, and indigo carmine), partial lifting was observed around the scar at the distal margin, suggesting the presence of UC-related fibrosis ( Fig. 1 **b** ). A circumferential mucosal incision and initial submucosal entry were performed using a Dual J knife. Then, robotic arm-assisted ESD traction was applied to facilitate dissection ( Fig. 1 **c** ) Robotic traction provided on-demand traction and countertraction with excellent exposure of the submucosal layer, enabling en bloc resection ( Fig. 1 **d** ). The resected specimen measured 28 × 30 mm and was retrieved. The total ESD time was 79 minutes, including scope exchange for device attachment. The post-ESD defect was completely closed with clips. No perforation or delayed bleeding occurred.

Endoscopic submucosal dissection using robotic arm-assisted lesion traction.Video 1

Robotic-arm traction effectively assisted ESD with stable and dynamic traction. It may facilitate dissection in the area with fibrosis and poor lifting with variable on-demand traction offered by the robotic system with multi-degree of freedom.

Endoscopy_UCTN_Code_TTT_1AQ_2AD_3AD
